# On the origin of the functional versatility of macrophages

**DOI:** 10.3389/fphys.2023.1128984

**Published:** 2023-02-23

**Authors:** Adam Bajgar, Gabriela Krejčová

**Affiliations:** ^1^ Faculty of Science, Department of Molecular Biology and Genetics, University of South Bohemia, Ceske Budejovice, Czechia; ^2^ Biology Centre, Institute of Entomology, Academy of Sciences, Ceske Budejovice, Czechia

**Keywords:** *Dictyostelium*, acanthamoeba, *Drosophila*, plasmatocytes, archaeocytes, *Porifera*, macrophage polarization, origin of macrophages

## Abstract

Macrophages represent the most functionally versatile cells in the animal body. In addition to recognizing and destroying pathogens, macrophages remove senescent and exhausted cells, promote wound healing, and govern tissue and metabolic homeostasis. In addition, many specialized populations of tissue-resident macrophages exhibit highly specialized functions essential for the function of specific organs. Sometimes, however, macrophages cease to perform their protective function and their seemingly incomprehensible response to certain stimuli leads to pathology. In this study, we address the question of the origin of the functional versatility of macrophages. To this end, we have searched for the evolutionary origin of macrophages themselves and for the emergence of their characteristic properties. We hypothesize that many of the characteristic features of proinflammatory macrophages evolved in the unicellular ancestors of animals, and that the functional repertoire of macrophage-like amoebocytes further expanded with the evolution of multicellularity and the increasing complexity of tissues and organ systems. We suggest that the entire repertoire of macrophage functions evolved by repurposing and diversification of basic functions that evolved early in the evolution of metazoans under conditions barely comparable to that in tissues of multicellular organisms. We believe that by applying this perspective, we may find an explanation for the otherwise counterintuitive behavior of macrophages in many human pathologies.

## Introduction

The human body is made up of more than two hundred types of cells (Castillo-Armengol et al., 2019). Unlike most cell types, macrophages display a striking level of functional versatility and an extraordinary degree of autonomy (Locati et al., 2020).

Macrophages represent the front line of the immune system, responsible for the recognition, phagocytosis, and elimination of pathogens and to control the inflammatory response by instructing other branches of the immune system *via* cytokine signaling (Cole et al., 2014). However, macrophage function is not limited to protection against foreign organisms. Macrophages are also involved in many homeostatic processes in the body (Biswas and Mantovani, 2012; Theret et al., 2019). Every day, millions of cells die in the human body and the constant substitution of cells and reconstitution of the extracellular matrix (ECM) governed by macrophages is fundamental for the health of any tissue in the body (Kwon et al., 2019; Batista-Gonzalez et al., 2020; Sender and Milo, 2021; Witherel et al., 2021).

Macrophages exhibit many specific characteristics predisposing them to be highly effective in the above functions. Macrophages are highly motile and crawl through the organism toward the site where they are needed (Xuan et al., 2015). Once in place, macrophages are sensitive to external signals and respond according to external conditions (Lavin and Merad, 2013). Their functional repertoire includes engulfing pathogens and removing damaged, senescent, or apoptotic cells. Internalized cellular material is processed and metabolically degraded in the phagolysosome. To this end, macrophages exhibit many specific metabolic pathways for processing and interconversion of phagocytosed organic material. In addition to sensing external signals, macrophages also excel in the production of a broad spectrum of signaling factors. Macrophages are central producers of cytokines in the body and are actively involved in interorgan signaling and regulation of homeostasis in healthy and pathological conditions (Arango Duque and Descoteaux, 2014).

The ability of macrophages to perform such a wide repertoire of functions is largely due to their metabolic plasticity. Sentinel macrophages typically reside in a quiescent state, referred to as M0, which serves as a baseline metabolic profile. From this state, macrophages can undergo metabolic polarization into various forms in response to different stimuli. Thus, various external factors trigger a specific macrophage expression program that leads to the modulation of major metabolic pathways to generate sufficient energy and specific metabolites required for an adequate functional response (Galvan-Pena and O'Neill, 2014). Therefore, metabolic polarization allows macrophages to adopt a specific functional polarization phenotype and perform unique functions efficiently (Liu et al., 2021). It was originally described that macrophages adopt two polarization phenotypes, defined as bactericidal (also known as pro-inflammatory; classically activated or M1) or healing (also known as anti-inflammatory; alternatively activated or M2) (Viola et al., 2019). However, more recent research has revealed many divergences from the polarized M1 and M2 types, such as metabolically activated macrophages (MMe) or macrophages activated by oxidized phospholipid (Mox) (Coats et al., 2017). Currently, the prevailing view is that M1 and M2 macrophages represent the two extremes of the entire continuum of all possible polarization phenotypes.

In addition to the general pro-inflammatory and homeostatic functions common to all macrophages, the population of tissue-resident macrophages found in virtually all tissues of the human body often perform highly specialized tasks (Nobs and Kopf, 2021). Among many others, some of the most well-studied tissue-resident macrophages include Kupffer cells in the liver, and microglia in the central nervous system, alveolar macrophages in the lungs, Langerhans cells in the skin, or peritoneal and adipose tissue macrophages (Wu and Hirschi, 2021). The progenitors of these tissue-resident macrophages migrate to destination tissue during embryonic development and their populations are sustained throughout the life of the individual by self-replication (Davies et al., 2013; Munro and Hughes, 2017). Tissue resident macrophages are functionally shaped by signaling factors characteristic for their particular tissue environment and exhibit distinct functional and morphological phenotypes. The role of tissue-resident macrophages ranges from fundamental functions, such as antibacterial responses and removal of dead and senescent cells, to advanced functions, such as promoting stem cell proliferation, regulating local and systemic metabolism, promoting lipid metabolism and thermogenesis, controlling sinoatrial node action potential, governing hematopoiesis, regulating synaptic pruning, inducing vascularization, and removing amyloid plaques and other potentially harmful substances from the extracellular space (Gordon and Plüddemann, 2017).

From the preceding paragraphs, it is clear that the mononuclear phagocyte system represents a central system for maintaining homeostasis that controls many physiological processes. However, the role of macrophages in the organism is not beneficial in all circumstances, and macrophages also play a significant role in the induction of several pathological conditions (Sica et al., 2015).

Macrophages may become inadequately activated in response to external stimuli, resulting in behavior that may appear counterintuitive in certain situations (Parisi et al., 2018). Excessive production of pro-inflammatory factors or excessive deposition of ECM components often leads to tissue and organ dysfunction and progressive development of pathology.

Excessive pro-inflammatory macrophage polarization is typically observed in obesity, non-alcoholic fatty liver disease, atherosclerosis, and neurodegenerative diseases (Lauterbach and Wunderlich, 2017; Mammana et al., 2018; Barreby et al., 2022). Likewise, chronic adoption of M2 macrophage polarization is associated with liver fibrosis, chronic obstructive pulmonary disease, Alzheimer’s disease, or cancer (Wang L. et al., 2019; Zhou et al., 2020). Pathologies in which macrophage activation plays a critical role are not limited to those listed here. In fact, lack of macrophage polarization plasticity in any tissue inevitably progresses to pathology. Nevertheless, the rationale behind the switch from the primarily protective role of macrophages to induction of pathology remains largely undetermined.

Fascinated by the functional versatility of macrophages, we seek to understand why macrophages have such an unusual degree of autonomy and responsibility. Understanding the evolutionary origins of macrophages may provide insight into how they have acquired critical properties necessary for their protective and homeostatic roles.

To reveal the origin of macrophages and their functional versatility, we decided to trace the characteristic features of mammalian macrophages back in the evolution of the animals. While investigating the origin of macrophage-like cells in the animal phyla, we realized that macrophage-like amoebocytes are present in virtually all multicellular animals.

We surmise that macrophage functional versatility reflects the ancient origin of these cells in free-living unicellular animals and that macrophage functional repertoire has further expanded with the emergence of multicellularity and the increasing complexity of the body plan of multicellular animals.

Information regarding unicellular animals and the emergence of multicellular animals is fragmented and can be inferred only from indirect evidence. Therefore, we decided to investigate the functional analogy between mammalian macrophages and free-living predatory amoeba (*Acanthamoeba*; Protists) (Tice et al., 2016). We then combined this with knowledge from the clades represented by unicellular animals (*Choanoflagellatea*, *Filasterea*, *Ichtyosporea*; *Holozoa*) (Hehenberger et al., 2017) to formulate an idea of what functions may have already been present in the unicellular free-living ancestor of animals.

We next set out to compare the characteristic features of mammalian macrophages with those observed in a social facultative multicellular amoeba (*Dictyostelium discoideum*; *Amoebozoa*) (Romeralo et al., 2011) to explore the possibility that the emergence of multicellularity has gone along with the expansion of the functional repertoire of macrophage-like amoebocytes.

Following this idea, we compare the functions known in mammalian macrophages with those observed in macrophage-like amoebocytes in sponges (*Porifera*; *Holozoa*), which represent multicellular animals without yet fully differentiated tissues and organs (Nielsen, 2019) and can thus provide some indication of what functions might be present in macrophage ancestors at the emergence of multicellular organisms.

**FIGURE 1:**
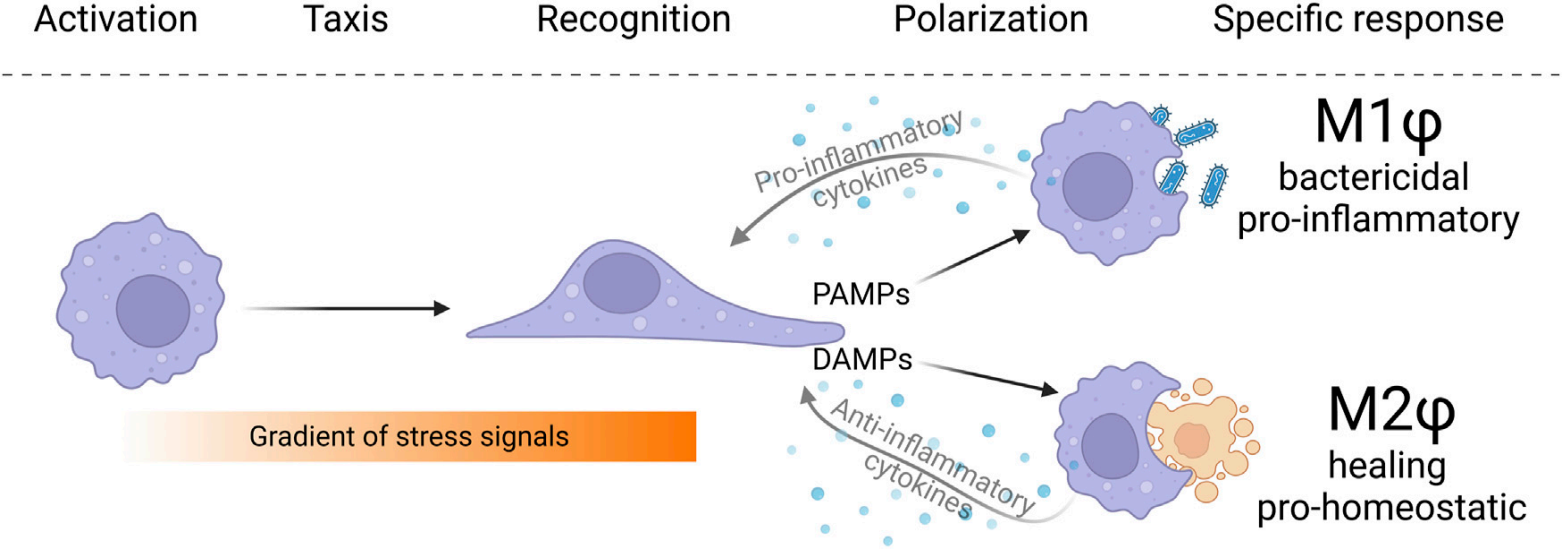
The typical macrophage behavior in tissues can be split into several consecutive phases. Macrophages first perceive activation signals indicating changes in tissue homeostasis. Subsequently, macrophages migrate against the concentration gradient toward the source of activating signals. Macrophages infiltrating disharmonious tissue are exposed to local signals in the form of pathogen-associated molecular patterns (PAMPs), danger-associated molecular patterns (DAMPs), and a cocktail of pro-inflammatory and anti-inflammatory cytokines. Based on extrinsic cues and intrinsic predetermination, macrophages adjust their metabolic setup and functionally polarize to M1 and M2 polarization phenotypes (in a simplistic view of the problematics). Macrophages, in an effort to resolve a stressful situation, engulf and eliminate pathogenic bacteria, or remove senescent and dysfunctional cells to restore tissue homeostasis.

Subsequently, we analyzed the characteristics of primitive macrophage-like plasmatocytes in the fruit fly (*Drosophila melanogaster*; *Metazoa*, *Insecta*) as a representative of a simple animal with fully developed tissues and organs at a level of complexity comparable to that of mammals (Cheng et al., 2018). For a historical perspective on the early discoveries of macrophage functional variability, see Box 1. The phylogenetic relationship of the compared clades and lineages is shown in the Figure 1.

BOX 1 **Metchnikoff’s predictions and the discovery of macrophages**

More than a century has passed since Metchnikoff formulated his theory of phagocytosis as the central mechanism of the immune response, for which he was awarded the Nobel Prize (Kaufmann, 2008). The attention this hypothesis attracted in the scientific community has unfortunately overshadowed many of the other postulates Metchnikoff made regarding the function of macrophages in the body. These speculations become particularly interesting in light of current knowledge about the function of macrophages, which goes far beyond their bactericidal function in the organism (Tauber, 2003).

Metchnikoff discovered the immune role of macrophages when studying the function of mesodermal amoeboid cells moving freely in the body of primitive multicellular organisms. In doing so, he paid close attention to the role these cells play in nutrient acquisition in organisms that do not have a digestive cavity and identified how these cells shape multicellular organisms during evolution and ontogeny (Merien, 2016). Metchnikoff proposed that complex multicellular organisms are inherently disharmonious and that macrophages induce physiological inflammation to achieve a harmonious whole (Tauber, 2017).

Metchnikoff’s exceptional observational skills and work ethic led him to recognize the importance of macrophages in maintaining nutritional, metabolic, and tissue homeostasis more than a century before the confirmation of this phenomenon by current molecular biological research.

## Macrophage versatility is based on a few fundamental macrophage features

Although macrophages perform many functions in the body, their behavior can be divided into several basic features that make them distinctly different from all other cells in the body. Generally, macrophages reside in the tissue in a quiescent state and calmly perceive signals from the environment (Holt and Grainger, 2012). Macrophages are equipped with a number of receptors for the recognition of chemoattractants and signaling substances that originate from indisposed cells and tissues, other immune cells, or produced by bacterial pathogens as their secondary metabolites. Most of the receptors recognizing the chemoattractant signals belong to the class of G-protein coupled receptors (GPCRs), such as formyl peptide receptor, folate receptor, adenosine receptor, purinergic receptors, and various chemokine receptors (Kim, 2018; O’Callaghan et al., 2021).

Upon chemokine recognition, the GPCR activates intracellular signaling that constitutes G-protein and arrestin as second messengers and leads to the activation of common stress response-related signaling cascades, such as PKC, PI3K-Akt, MAPK-ERK, AP, JAK-STAT, etc. The induced transcriptional program leads to increased cytoskeleton reorganization, cell shape changes, directed motility, secretion of lysosomal enzymes, phagocytosis, and activation of the respiratory burst (Wang X. et al., 2019).

Macrophages are chemotactically guided through the environment against the concentration gradient of extracellular chemical stimuli, such as chemokines, polyunsaturated fatty acid metabolites (leukotrienes and eicosanoids), components of the complement cascade (C3a, C5a), or formyl peptides (Sokol and Luster, 2015). Unlike most cell types in the mammalian organism, macrophages exhibit active migration, facilitated by rapid remodeling of the actin cytoskeleton. Macrophages primarily use two distinct types of migration, namely amoeboid and mesenchymal. Amoeboid migration is a rapid movement driven by an actin-rich pseudopod at the leading edge, hydrostatically generated blebs, and a highly contractile uropod at the trailing edge. This movement is characterized by weak or absent adhesion to the substrate and low-level proteolysis of the ECM. In contrast, mesenchymal movement is characterized by cell adhesion to the substrate *via* integrins, cadherins, or fibronectins and requires enzymatic disruption of binding to the ECM (Pizzagalli et al., 2022).

To effectively distinguish various pathogens from the body’s own cells, macrophages must sense and recognize specific pathogen-associated antigens on the surface of the foreign cells. These molecules are recognized by immune-cell-specific receptors called pattern recognition receptors (PRRs) (Amarante-Mendes et al., 2018). Mammalian macrophages exhibit a wide spectrum of PPRs, categorized into several classes according to their structure. Many of these receptors, such as toll-like receptor family, scavenger receptors, c-type lectins, or NOD-like receptors, are evolutionarily ancient, and their ability to recognize pathogen-associated molecular patterns (PAMPs) has been shaped over the billions of years of coevolution between pathogen and host (Li and Wu, 2021). Antigen binding to PRR activates macrophage immune-related cascades, such as NFĸB, ERK, JNK, and p38, which initiate complex signaling cascades that allow remodeling of the macrophage cytoskeleton and formation of membrane invaginations to engulf the particle and form a phagosome. Subsequently, the primary phagosome fuses with acidic lysosomes, which contain a mixture of enzymes that cleave the phagocytosed material. During the respiratory burst, the NADPH oxidase NOX2 pumps massive amounts of reactive oxygen species (ROS) into the phagolysosome to destroy its contents. Elimination of pathogenic bacteria is enhanced by the activity of natural resistance-associated macrophage proteins (NRAMP) transporters, which pump divalent ions onto the phagolysosome lumen. Additionally, macrophages polarize toward a pro-inflammatory state, releasing a mixture of pro-inflammatory cytokines and opsonizing factors (Mogensen, 2009).

The underlying mechanism enabling these changes is the modification of cellular metabolism. Strikingly, pro-inflammatory macrophages adopt aerobic glycolysis as the predominant method of ATP production, driven by the stabilization of the transcription factor HIF1α (Wang et al., 2017). While oxidative phosphorylation in mitochondria generates significantly more ATP per glucose molecule, M1 macrophages favor aerobic glycolysis, likely due to the rate of ATP production. In addition, aerobic glycolysis allows increased NADPH production in the pentose phosphate pathways, which is used as a building block for many biomolecules. Since pyruvate is converted to lactate by lactate dehydrogenase and excreted from the cell, the TCA cycle is supplemented with glutamine causing it to be “interrupted” or “rewired”. As a result, TCA cycle intermediates accumulate and contribute to further stabilization of HIF1α. At the same time, mitochondria, which are liberated from generating ATP in oxidative phosphorylation, instead generate ROS by the reversed electron flux at the respiratory chain complex1 (Viola et al., 2019). M1 polarization is also characterized by the specific utilization of arginine, which is converted by L-arginase to citrulline, and growth-inhibiting NO, which is transported to the phagolysosome (Palmieri et al., 2020). M1 polarization is associated with the production of pro-inflammatory cytokines, such as IL-1, IL-6, or INFγ, which further inform other cells of danger (Nonnenmacher and Hiller, 2018). Once the pathogen is eliminated, the immune response is not yet complete, M2 macrophages need to be recruited to promote the resolution of inflammation and restore homeostasis.

In addition to pathogenic activation, macrophages are activated by signals produced by damaged, metabolically stressed cells and tissues, known as DAMPs (danger-associated molecular patterns), leading to M2 macrophage polarization (Ferrante and Leibovich, 2012). While the functions of M1 macrophages are relatively simple, the functions of M2 macrophages are more diverse. The main goals of M2 macrophages are to resolve inflammation, protect against viral and fungal infections, promote angiogenesis, facilitate ECM remodeling, support tissue healing, and regeneration, and remove senescent and damaged cells by efferocytosis (Wang L. et al., 2021). One of the important properties of M2 macrophages is the maintenance of immunological tolerance, i.e., the prevention of an immune reaction against host antigens. Thus, their function is particularly crucial in organs that must tolerate foreign antigens, such as those of the developing fetus or developing spermatids in the testis (Porta et al., 2009). This tolerogenic property also allows the presence of symbiotic bacteria. However, excessive adoption of M2 macrophage polarization may become detrimental as it induces tissue fibrosis, leading to chronic infections and promotion of tumor cell growth (Lin et al., 2019).

M2 macrophages differ significantly from their M1 counterparts in cellular metabolism, which determines their different function. While the amino acid arginine serves as a substrate for iNOS in M1 macrophages, as it is essential for the production of ROS (Rodriguez et al., 2017), M2 macrophages primarily use arginine as a substrate for arginase, promoting its conversion to ornithine and urea. Ornithine is subsequently used as a substrate for forming ECM components, making M2 macrophages essential contributors to tissue regeneration and wound healing (Szondi et al., 2021). Hence, after the elimination of pathogenic invaders, pro-inflammatory macrophages are gradually replaced by M2 macrophages, which trigger the regeneration of the wounded tissue and promote vascularization, ECM synthesis, and inflammation resolution. In addition, M2 macrophages participate in ECM remodeling by producing matrix metalloproteases, cathepsins, and other enzymes that reorganize collagen fibers and by modulating fibroblast function (Witherel et al., 2021).

M2 macrophages are also responsible for maintaining tissue homeostasis under physiological conditions by detecting and removing apoptotic and damaged cells through efferocytosis. The term “efferocytosis” was introduced by deCathelineau and Henson, (2003) to describe the phagocytosis of apoptotic cells. Unlike phagocytosis of foreign objects, which triggers inflammation and antigen presentation, efferocytosis of apoptotic cells upregulates anti-inflammatory cytokines and compounds promoting tissue healing. During efferocytosis, macrophages are guided chemotactically to apoptotic and senescent cells through the detection of “find me” signals, such as nucleotides (ATP, ADP, or UDP), lysophosphatidylcholine, or sphingosine-1-phosphate (Ravichandran, 2010). The receptors responsible for recognizing apoptotic cells differ from those involved in phagocytosis. Subsequently, macrophages respond to “eat me” signal molecules, such as phosphatidylserine, oxidized phospholipids, DNA, or annexin A1, exposed on the surface of the cells destined for efferocytosis. While the engulfment process resembles macropinocytosis, the machinery fusing the efferosome with the lysosome is analogous to phagolysosome formation. Therefore, M2 macrophages exhibit a wide spectrum of enzymes that can metabolize phospholipids and DNA fragments and neutralize otherwise dangerous modified lipids and proteins (Martin et al., 2014).

M2 macrophages can be divided into different polarization subtypes, such as M2a, M2b, M2c, and M2d, based the on the specific cocktail of chemokines, cytokines, and growth factors they polarize with and subsequently produce (Ross et al., 2021). In addition, plethoras of polarization phenotypes have also been described in the context of hypertrophic adipocytes or atherosclerotic plaques. For example, ingestion of heme by macrophages leads to the adoption of the Mhem polarization phenotype, internalization of hemoglobin to M (Hb), and the exposure of oxidized lipids to Mox (Lin et al., 2021). Since these macrophage subsets are often characterized only by mammalian-specific surface markers and do not exhibit a characteristic functional profile, tracking them during evolution is impossible (Natoli and Monticelli, 2014). For this reason, in this paper, we focus only on the functionally well-characterized macrophages M1 and M2 as representatives of the two phenotypic extremes.

**FIGURE 2:**
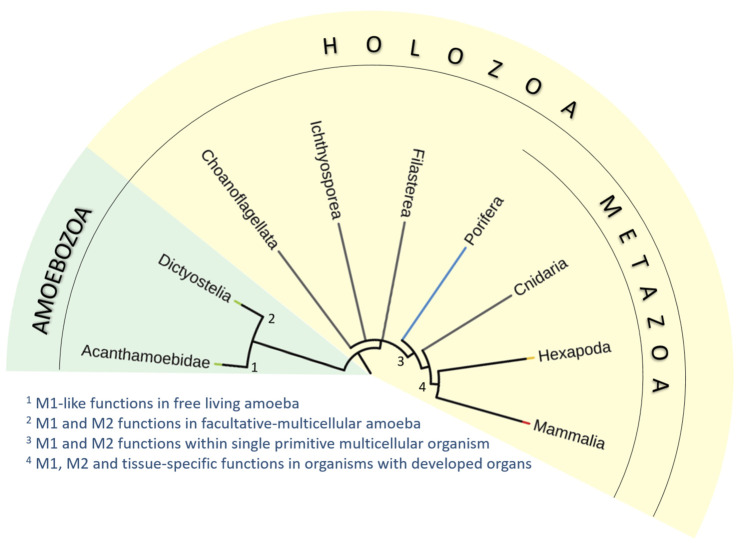
Simplified phylogenic tree of *Holozoa* and their relatives. We have analyzed the occurrence of characteristic features of mammalian macrophages in unicellular free-living amoeba (*Acanthamoeba*; *1*), social facultative-multicellular amoeba (*Dictyostelium*; *2*), macrophage-like amoebocytes in primitive multicellular animal lacking true tissues and organs (*Porifera*; *3*), and macrophage-like plasmatocytes of simple multicellular organisms with fully developed organs and tissues (*Drosophila*; *4*). We propose that many macrophage characteristics are inherited from unicellular ancestors of animals. The functional repertoire of macrophages then diversified with the emergence of multicellularity and increasing complexity of body plan and development of organ systems.

Overall, macrophages are truly unique cells of the animal body that can play many roles in different tissues and body contexts by combining several specific properties. In particular, macrophages are exceptional at sensing chemotactic signals, exhibiting controlled active motility, recognizing molecular patterns associated with pathogen or tissue damage, and adopting metabolic and functional polarization accordingly. These properties predispose them to deal with stressful situations in the body (Figure 2).

## Macrophage functional repertoire has expanded with the increasing complexity of the animal body

### Free-living predatory amoebas share many similarities with M1 macrophages

By comparing the characteristics of mammalian macrophages with the prey-hunting strategies of free-living amoebae, we can find surprising similarities. *Acanthamoeba* and macrophages share the principal mechanisms used for chemotaxis towards bacteria, motility, interaction with bacteria, phagocytosis, the killing of bacteria in the phagolysosome, and production of antimicrobial peptides (Siddiqui and Khan, 2012a).

The underlying molecular mechanisms show a remarkable degree of similarity, documented by the fact that human intracellular pathogens use the same strategies to escape the bactericidal mechanism in the macrophage and *Acanthamoeba* (Molmeret et al., 2005). Therefore, *Acanthamoeba* is often viewed as a training ground for microbial organisms to become successful human and animal pathogens and a melting pot for horizontal gene transfer between different bacterial strains (Salah et al., 2009).

*Acanthamoeba* is a free-living heterotrophic Protist that specializes in hunting microbes for its nutritional needs. *Acanthamoeba* has two life stages; an active trophozoid or a dormant double-walled cyst, which can withstand adverse environmental conditions for long periods of time (Siddiqui and Khan, 2012b). In terms of life strategy, *Acanthamoeba* as professional phagocytic bactericidal omnivores do not differ significantly from the basal groups of *Holozoa* and are not expected to substantially differ from unicellular ancestors of animals (Lang et al., 2002).

Immediately, we can discern similarities between *Acanthamoeba* and mammalian macrophages with respect to size, behavior, cellular ultrastructure, and chemical composition (Rayamajhee et al., 2022). Like mammalian macrophages, *Acanthamoeba* can sense chemical signals from the environment and approach the signal source by chemotaxis through motility based on actin and myosin remodeling (Swart et al., 2018). In-depth studies of chemotactic factors have identified various bacterial metabolic products, such as formyl-methionyl-leucyl-phenylalanine, lipopolysaccharide, lipoteichoic acid, cAMP, lipid A, or N-acetylglucosamine. In analogy to mammalian macrophages, the perception of chemotactic signals in *Acanthamoeba* is mediated *via* GPCRs (Schuster and Levandowski, 1996). Most of these signals are products of bacterial metabolism or fragments of surface bacterial macromolecules and also serve as potent chemoattractants for mammalian macrophages and neutrophils (Nadesalingam et al., 2005).

A detailed study of crawling in free-living amoebae revealed that the migratory mechanisms used by macrophages and amoebae are identical (Campolo et al., 2021), indicating their ancient origin in the common ancestor of *Amoebozoa* and *Opisthokonta*.

Once macrophages approach the site of origin of chemotactic signals, they must recognize which cells are to be engulfed and eliminated in the phagolysosome. Many of the receptors used by macrophages to recognize pathogenic bacteria can also be found in some form in *Acanthamoeba*. For instance, the C-type lectin mannose receptor, which is abundantly expressed by mammalian macrophages, is used by *Acanthamoeba* to identify prey and engulf it (Allen and Dawidowicz, 1990).

*Acanthamoeba* recognizes and binds the bacteria, and the subsequent processes of phagocytosis and destruction of the pathogen show a high degree of similarity to mammalian macrophages. Pathogen recognition leads to massive reorganization of F-actin filaments in both macrophages and *Acanthamoeba*, resulting in dynamic probing, disruption of the cortical F-actin layer, nucleation and polymerization of F-actin filaments, phagosome closure, and particle internalization (Bowers, 1977; Alsam et al., 2005). Internalized bacteria are inactivated and enzymatically processed in the phagolysosome. Ultrastructural analysis of *Acanthamoeba* revealed that they contain many lysosomes containing a cocktail of degradative enzymes (Alsam et al., 2005; Salah et al., 2009). After fusing the phagosome with the lysosome, V-ATPases embedded in the phagolysosomal membrane pump hydrogen ions inside the phagolysosome to acidify the phagolysosomal lumen (Akya et al., 2009). The bacteria are then exposed to superoxide ions and hydrogen peroxide, in a process called oxidative burst. The active form of oxygen is produced in the lumen of the phagolysosome by NADPH oxidase activity, supported by altered mitochondrial metabolism (Rayamajhee et al., 2022). To further inhibit the ability of bacteria to avoid the phagolysosome, additional transporters are housed in the phagolysosomal membrane. NRAMPs transport sequestered divalent ions (Mn^2+^, Fe^2+^, Zn^2+^, and Cu^2+^) outside the phagolysosomes, thereby limiting the ability of engulfed bacterial to use metalloenzymes required to escape the phagolysosome (Siddiqui et al., 2019).

Overall, mammalian macrophages and *Acanthamoeba* display striking similarities in the molecular mechanisms involved in directional motility, recognition, binding, engulfment, and phagolysosome processing of bacteria.

To get a better idea of the characteristics of the last unicellular common ancestor of animals, we can compare the genomic information of primitive multicellular animals with their unicellular relatives forming the basal clades in a phylogenetic tree of *Holozoa*, such as *Filasterea*, *Ichthyosporea*, and *Choanoflagellata* (Ros-Rocher et al., 2021). We can assume that the genes shared by these groups were already present in the unicellular ancestors of modern multicellular animals. Therefore, we can expect that the last unicellular ancestor of animals already possessed a wide repertoire of genes required for multicellularity, such as molecules for intercellular adhesion, communication, and interaction with the ECM (King, 2004). We speculate that many of these genes are analogous to those characteristically used by macrophages to carry out similar functions.

Given that most of the characteristic features of macrophages observed in *Acanthamoeba* are associated with bacterial recognition, endocytosis, and elimination, we hypothesize that these abilities later evolved into a protective bactericidal function as part of the host immune response in multicellular animals (Hartenstein and Martinez, 2019). This suggests that the evolutionary origin of the bactericidal function of mammalian macrophages arose prior to the branching of *Amoebozoa* and *Opisthokonta,* most likely in the environment of a free-living unicellular amoeboid cell.

Moreover, this implies that the features underlying the function of M1 bactericidal macrophages represent an ancestral macrophage phenotype and that M2-like macrophage features arose later in evolution, potentially coinciding with the emergence of multicellularity, as discussed in the following paragraphs.

### Macrophage homeostatic features arose along with multicellularity

Based on comparisons of macrophages with free-living predatory *Acanthamoeba* and basal unicellular relatives of metazoans, we hypothesize that many specific features of macrophages associated with their bactericidal function derive from unicellular animal ancestors. However, *Acanthamoeba* does not possess analogous homeostatic, regulatory, and metabolic functions as mammalian macrophages. We, therefore, explore the possibility that the functional repertoire of macrophages has expanded substantially with the evolution of multicellularity.

We explore the analogy between the features observed in mammalian macrophages and *D. discoideum*, a close relative of *Acanthamoeba,* used as a model organism to investigate facultative multicellularity (Bozzaro, 2013). *Dictyostelium* possesses a complex life cycle. Typically, *Dictyostelium* resides in the vegetative state of free-living haploid amoebae that divide periodically by mitosis and prey on microbes for nutrition. When food becomes scarce, starving vegetative amoebae enter a social life form, or a sexual cycle. During the social cycle, the amoebae aggregate to form a multicellular pseudoplasmodium (also known as a slug). The slug conforms to all the parameters of a multicellular organism. The originally amoeboid vegetative cells differentiate into four distinct cell types that coordinate their behavior and give rise to a fruiting body that produces resistant spores (Flowers et al., 2010).

In terms of their biology, the vegetative cells of *Dictyostelium* resemble the trophozoids of *Acanthamoeba*. Therefore, it is not surprising that, like *Acanthamoeba*, vegetative cells of *Dictyostelium* also share many features with mammalian pro-inflammatory macrophages (Bozzaro and Eichinger, 2011). Observations from *Dictyostelium* vegetative cells conveniently complement our previous statements, discussed in the following paragraph. Interestingly, despite the vegetative cells of *Dictyostelium* being freely motile, we can observe a certain degree of sociality. The behavior of these vegetative amoebae is coordinated by mutual communication of soluble signaling factors, which could provide the basis for the later emergence of cytokine signaling in macrophages. In fact, similar communication has been observed in *Acanthamoeba* (Golé et al., 2011).

Vegetative cells of *Dictyostelium* perceive signals from their environment and localize bacteria as a nutrient source through a gradient of their secondary metabolites, such as folic acid, retinoic acid, lipopolysaccharides, and lysophosphatidic acid (Iglesias, 2012). The perception of these chemotactic signals is mediated by GPCRs (e.g., folic acid receptor, retinoic acid receptor) that trigger strong chemotaxis and foraging behavior (Iglesias, 2012). Recently, it was shown that vegetative *Dictyostelium* cells are also attracted to signaling factors of a non-biological nature. Exposure of vegetative cells to a gradient of Mg^2+^, Zn^2+^, or hydrogen peroxide induces high chemotactic motility (Consalvo et al., 2019). Most factors that activate vegetative amoebae of *Dictyostelium* also have a strong activating and chemotactic effect on mammalian macrophages (Cammer and Cox, 2014). This is consistent with the observation that the vast majority of receptors carried by vegetative cells of *Dictyostelium* are retained in mammalian macrophages. Indeed, exposure of macrophages to the chemoattractants mentioned above leads to increased macrophage motility (Xu et al., 2021).

*Dictyostelium* is equipped with a wide spectrum of receptors that recognize pathogens and other cells to be engulfed, which are classified as (PRRs). These surface receptors show substantial homology to many mammalian PRRs, such as scavenger receptors (LIMP-2), toll-like receptors (tirA, tirB), leucine-rich repeats receptors (LrrA), and C-type lectin receptors. Activation of these receptors triggers intracellular signaling cascades initiating phagocytosis, phagosome maturation and bacterial killing, and stress-related cascades and detoxification response (Dunn et al., 2018).

The process of F-actin remodeling and phagolysosome formation starts with the activation of one of the GPCRs. For example, activation of the folate receptor or the homolog of the toll-like receptor tirA leads to activation of conserved RAS-PI3K and ERK-MAPK signaling, resulting in induction of actin polymerization, increased motility and phagocytosis (Chen et al., 2007). Actin nucleation and branching are mediated by actin remodeling complexes consisting of WASp Arp2/3 and SCAR/WAVE proteins (Vogel et al., 1980). The mechanism described above in *Dictyostelium* resembles that observed in mammalian macrophages, in which activation of surface toll-like receptors (TLRs) or Fc receptors analogously initiates increased motility, phagocytosis, and production of pro-inflammatory factors (Schmitz et al., 2004). The detailed mechanism of phagolysosome maturation in *Dictyostelium* is now well described (Cosson and Lima, 2014). Interestingly, this mechanism is principally homologous to that of mammalian macrophages. Internalized bacteria are eliminated in the phagolysosome by the sequestration of divalent ions by the activity of NRAMT transporters and by ROS production by the mitochondrial NADH-dependent oxidase NOX2 (Lardy et al., 2005). Maturation of the phagolysosome containing indigestible bacterial remnants leads to their exocytosis and neutralization of the phagolysosome. Alternatively, ingested bacterial remnants are processed by autophagy, which is particularly important during starvation and infection by intracellular pathogens (Mesquita et al., 2017). The remarkable analogy of these processes between *Dictyostelium* and the mammalian macrophage rule out the possibility of convergent evolution and further supports the adoption of features characteristic of bactericidal macrophages already in our unicellular ancestors. In general, many features of vegetative amoebas of *Dictyostelium* resemble those observed in M1 mammalian macrophages.

In certain situations, vegetative amoeboid cells can switch from unicellular to multicellular life. Amoeboid vegetative cells constantly coordinate cell growth and division through signals that inform each other about their density and nutrient availability (Loomis, 2014). Nutritionally supplied cells continuously produce prestarvation factor (PSF), which inhibits cell behavior leading to aggregation. When PSF production decreases due to nutrient deficiency, cells begin to produce conditioned medium factor (CMF), which triggers the release of a pulse of cAMP. The cAMP signal is further amplified by surrounding cells, creating a concentration gradient that allows aggregation (Clarke and Gomer, 1995).

The cellular cascade that transduces the extracellular cAMP signal is of particular interest. Extracellular cAMP binds to the G-protein-coupled chemoattractant receptor cAR1, which serves as a docking receptor for *β*-Arrestin. This interaction triggers signaling through second messengers well known from mammalian cells, such as GSK3, ERK, Ras/GTP, and PI3K, and leads to activation of the effectors PKB, PKA, STAT, and TORC2, which drive an expression program controlled by the GATA family transcription factors (Loomis, 2014; Singer et al., 2019).

The transition from the unicellular to the multicellular life stage is associated with significant transcriptomic changes. These changes are achieved primarily through the propagation of repressive epigenetic modifications that functionally shape amoeboid cells to become more cooperative. ATAC-seq. analysis of vegetative cells undergoing transition revealed that the most significantly enhanced genes are classified as factors regulating ECM organization, cell adhesion, differentiation, and morphogenesis (Wang S. Y. et al., 2021). Recently, it has been shown that alternation of mitochondrial metabolism is a prerequisite for adopting tolerogenic cell behavior and multicellularity (Glöckner et al., 2016; Singer et al., 2019; Kelly et al., 2021). This process highly resembles cAMP tolerogenic behavior of mammalian myeloid cells required for macrophages to perform tissue homeostatic tasks. (Sciaraffia et al., 2014).

When transitioning to the social phase of the life cycle, *Dictyostelium* cells inevitably encounter many problems common to multicellular animals, indicating an increased need for self-recognition and regulation. Previously, it has been described that the social life stage of *Dictyostelium* is associated with various types of cellular relationships, such as cheating and allocheating, but also altruism and self-sacrifice (Strassmann and Queller, 2011).

The multicellular body of the pseudoplasmodium consists of thousands of cells. Most of the cells in the body are destined to form future morphological structures of the sorocarp, such as stem cells, cup cells, and spores (Jang and Gomer, 2011). However, when tracing the evolution of macrophage-like features, a fourth subpopulation of sentinel cells deserves particular attention. Sentinel cells have protective, homeostatic, and regulatory functions and, therefore, resemble the primitive immune system of multicellular organisms. Sentinel cells are free-moving cells that phagocytose bacteria and toxins until they are eventually eliminated. Compared to other slug cells, sentinel cells show increased expression of the gene coding for *Toll-interleukin receptor domain-containing protein* (*tirA)*, which is analogous to the mammalian toll-like receptors (Brock et al., 2016a).

Sentinel cells protect the snail from potentially pathogenic bacteria by releasing extracellular DNA traps and producing ROS to the external space (Zhang and Soldati, 2016). In case of infection by intracellular bacteria, sentinel cells cleanse the slug of infected cells, keeping the rest of the organism healthy and giving rise to uninfected spores (Farinholt et al., 2019). In addition to their protective role, sentinel cells exhibit a high degree of tolerogenic behavior and can discriminate between genetically related and unrelated cells in aggregation (Hirose et al., 2011). Thus, in the multicellular stage of life, only closely related cells are nourished by sentinel cells. Indeed, their tolerogenic internal predetermination is represented by the rather unexpected observation that the multicellular stage of *Dictyostelium* can maintain commensal bacteria, in a specific form of farming for nutritional symbiosis (Brock et al., 2013; Brock et al., 2016b). By these features, the sentinel cells of the slug resemble the functions of mammalian M2 macrophages.

Collectively, the features observed in *Dictyostelium* cells during the transition from the unicellular to the multicellular life stage may provide critical insight into how macrophage-like features emerged with multicellularity in animals.

To explore this idea, we took inspiration from a study that compared the genomes of multicellular animals and their unicellular relatives to identify the genes present in the last common multicellular ancestor of animals which expanded upon the emergence of multicellularity (Ros-Rocher et al., 2021). Such genes are mostly related to intercellular signaling, signal transduction, adhesion molecules, and regulators of the cytoskeleton. Furthermore, multicellular animals also show an increase in the repertoire of transcription factors and genes mediating epigenetic modifications, suggesting the need for temporal functional plasticity and restriction of specific traits to certain subpopulations of cells in the multicellular body (Hinman and Cary, 2017; Herron et al., 2018).

As mentioned, we may assume that the transition to multicellularity is conditioned by several adaptations on various levels of regulation, including epigenetic remodeling, transcriptional programming, metabolism, and cell behavior. The most significant changes are related to enhanced expression of adhesive molecules, signaling factors, enzymes involved in remodeling of ECM, and adoption of tolerogenic predetermination.

Comparison of macrophage-like properties in unicellular vegetative amoebae and sentinel cells in multicellular slugs reveals a functional shift of macrophage-like properties, from clearly pro-inflammatory and bactericidal, to protective but also tolerogenic and regulatory. Furthermore, we speculate that many of the functions that arose in unicellular amoebae to hunt microbes were functionally repurposed and served as a solid basis for the evolution of multicellularity. For an overview of the evolving hypotheses concerning the primary cell type in animals, see Box 2.

BOX 2 **macrophages in perspective of emerging multicellularity**

The emergence of multicellular animals is a fascinating event in the evolution of metazoans. Formulation of the theory of common descent in the nineteenth century led many famous evolutionary and developmental biologists to seek a thorough explanation of what the hypothetical last common ancestor of all animals (the mysterious “Urmetazoan”) may have looked like (King, 2004). Among the most famous is Earnest Haeckel, whose theories suggested that the most ancestral animal cell was the amoeboid cell, which, under certain conditions, could have progressed to the colonial stage of life (Brunet and King, 2022). However, this theory was challenged by Elie Metchnikoff, who was convinced that the most ancestral animal cell was equipped with a flagellum, as is observed in basal groups of *Holozoa,* such as *Porifera* and *Choanoflagellata* (Brunet and King, 2022). However, Metchnikoff’s theory had major discrepancies, as it failed to explain the striking similarity between the amoeboid cells observed in animals and the unicellular *Protista*.

Recently, this obstacle has been resolved by the discovery that *Choanoflagellata* are able to switch to amoeboid cells under certain circumstances (Brunet et al., 2021). In addition, it has been found that amoeboid cells can give rise to all other cell types in *Porifera* (Müller, 2006). This suggests that amoeboid cells represent the most ancestral cell type in metazoans, and that the amoeboid cell type has been retained and is present throughout the metazoan phylogenetic tree, rather than being evolutionarily discontinued (Brunet and King, 2017). According to the current generally accepted theory, the ancestor of animals was a facultative multicellular organism that alternated cell types between free-moving social amoebae and a multicellular stage in which amoebocytes differentiate into collar containing flagellated cells (Brunet and King, 2017). As the complexity of multicellular organisms increased, as did the need for molecules responsible for cell colony cohesion, signaling, cell differentiation, and maintenance of homeostasis (Brooke and Holland, 2003; Grau-Bové et al., 2017).

### Macrophage-like amoebocytes perform both M1 and M2 features within *Porifera*

In the previous section, we described that in the multicellular stage of the social amoeba *D. discoideum*, subpopulations of sentinel cells retain features of professional phagocytes, and play a protective, regulatory, and homeostatic role in the pseudoplasmodium. This raises the question of whether the presence of amoeboid cells fulfilling these tasks is essential for the functioning of multicellular organisms. Virtually every known multicellular animal has a highly motile professional phagocyte that performs protective, healing, regenerative, regulatory, and homeostatic functions in the organism (Brunet et al., 2021).

We can gain a comprehensive understanding of the range of functions that professional phagocytes can perform in a primitive multicellular organism by studying sponges (*Porifera*), which represent a phylum of basal multicellular organisms with incomplete tissues and organ systems (Feuda et al., 2017). Members of *Porifera* phylogenetically represent the most ancestral metazoans. They are primitive multicellular heterotrophic organisms and represent the sister group of multicellular animals. These aquatic creatures depend on filtering water from which they obtain nutrients. Although they lack distinct tissues and organs, such as nervous, digestive, or circulatory systems, they possess several cell types with specialized functions (Thacker et al., 2014).

The structure of the sponge body is relatively simple. The body is formed by a gel-like, amorphous matrix called the mesohyl, sandwiched between two layers of cells, the outer pinacoderm and the inner choanoderm. The mesohyl is composed of ECM components commonly found in other animals, such as collagen, dermatopontin, galectin, and fibronectin-like glycoproteins (Dahihande and Thakur, 2021). Most sponges live a sedentary lifestyle and filter nutrients from the water using specialized cells called choanocytes. Choanocytes are equipped with flagella, whose movement creates water flow, and cilia, which form a filtering collar to trap food particles. The food particles are internalized by the choanocytes by nutritive phagocytosis and processed in food vacuoles (Laundon et al., 2019). Nutrients must then be distributed throughout the body, from choanocytes to other cell types. This function is performed by archaeocytes, which receive nutrients from choanocytes and transport them, by virtue of their high motility, throughout the mesophyll to the nutritionally demanding cells (Hartenstein and Martinez, 2019).

As already mentioned, the protective role of macrophages originates from the wild unicellular ancestors of animals, in which it evolved as a nutritional phagocytosis of bacteria. Choanocytes and archaeocytes are professional phagocytic cells in *Porifera*. The identity of these cells is not completely fixed and both cells can undergo a change to the opposite cell type under certain conditions. As such, it is difficult to distinguish which of these 2 cell types represents the ancestor of macrophages in bilaterians (Nakanishi et al., 2014). Since archaeocytes are freely motile and play a protective role in sponges, they show functional similarities to macrophages of bilaterians, therefore, it is feasible that archaeocytes represent the ancestors of these cells. The mechanism of nutrient uptake by choanocyte-like cells and nutrient distribution by freely motile amoebocytes is highly conserved in animals, with the exception of vertebrates and insects (Hartenstein and Martinez, 2019).

Archaeocytes, also called amoebocytes, are macrophage-like cells dispersed in the mesophyll of the sponge. Archaeocytes are unique from other sponge cells because they retain a significant degree of totipotency and can give rise to any other cell type. An isolated suspension of archeocytes can regenerate the entire body of sponges, suggesting that they represent their ancestral cell type (Ereskovsky et al., 2021).

Archaeocytes were originally described by Ellie Metchnikoff in 1892 and denoted as macrophages of the sponge by Van de Vyver more than a century later (Muller, 2003). Sponges are exposed to a many potential pathogens and foreign particles from filtering the water and need an effective system for their elimination (Dzik, 2010). Archaeocytes play a central role in the protection of sponges from pathogens. Sequencing of the *Porifera* genome revealed that sponges exhibit a broad spectrum of pathogen recognition receptors that are homologous to the main PRR groups found in mammals, such as GPCRS, NOD-like receptors, cysteine-rich receptors, scavenger receptors, and receptors from the immunoglobulin superfamily (Wiens et al., 2005; Srivastava et al., 2010). A recent study also documented the presence of the TLR-mediated signaling cascade (Germer et al., 2017).

Upon recognition of PAMPs, archaeocytes activate the signal transduction pathway in which MyD88 acts as a second messenger and activates effector transcription factors known in mammalian immune response, such as IRAK, TRAFs, and NFĸB (Müller et al., 2009). Activation of these immune-related pathways induces the production of galectins, perforins, and ROS as molecules participating in the opsonization of the pathogen and its elimination (Wiens et al., 2005). Until now, 39 different lectins have been identified in the genomes of the *Porifera* phylum, including C-type lectins, tachylectin-like, F-type lectins, and galectins (Gardères et al., 2015). Thus, archaeocytes, after their activation by pathogens, exhibit features, and behavior with a high degree of homology to mammalian proinflammatory macrophages.

However, many situations require an advanced level of coordination and tolerant behavior of archaeocytes. In the following paragraphs, we will discuss the indispensable role of archaeocytes in immune tolerance, healing, regeneration, self-identification, and reproduction.

Archaeocytes display surprising tolerogenic potential, as commensal bacteria do not invoke bactericidal behavior. However, the tolerogenic mechanism has not yet been satisfactorily elucidated (Maldonado, 2016; Carrier et al., 2022). Archaeocytes are also indispensable for healing and tissue regeneration (Boury-Esnault, 1977). During healing, the wound is infiltrated by archaeocytes and damaged cells are cleared from the local environment. Archaeocytes then secrete components of ECM and differentiate into other cell types, giving rise to the regular structure of the body. At his point, the archaeocytes may also phagocytose the grey cells, which contain large amounts of glycogen and osmiophilic inclusions and thus serve as a nutrient reservoir (Fernàndez-Busquets et al., 2002).

Sponges possess the ability of whole body regeneration, either from a body fragment or by aggregation of dissociated cells. After the cells of the sponge body are dissociated to a cell suspension, the cells dedifferentiate to amoebocyte morphotypes, and the archaeocytes represent the most abundant cell type in the suspension. Subsequently, the cells aggregate, presumably due to pseudopodial activity, and differentiate into the appropriate cell types to form the body of the sponge (Buscema et al., 1980). Strikingly, if the bodies of two distinct sponges are dissociated into single-cell suspension, the cells sort in a species-specific manner and the two individuals are eventually reconstituted. Moreover, archaeocytes are sufficient to reconstitute functional sponges without any other cell type (Lavrov and Kosevich, 2014). Hence, they represent the totipotent stem cells of the organism. These observations undeniably demonstrate Porifera’s ability to recognize its own genetically related cells from others.

Transplantation studies have further contributed to the understanding of this phenomenon. Whether a graft is accepted or rejected depends on the phylogenetic distance between the recipient and the donor. It has been shown that a graft comprised of cell from the same species and strain fuses with the recipient and is eventually accepted. Transplantation of an allograft causes the formation of a barrier between the transplanted tissues or a cytotoxic reaction at the graft interface, leading to the separation of the allograft cells (Smith and Hindeman, 1986). A small subset of cell types are involved in allograft rejection. Archaeocytes and lophocytes, which are recruited from the mesoglea and migrate along the border of both tissues, either phagocytose healthy donor cells to separate the tissues or exhibit cytotoxic activity to destroy cells in contact (Gaino et al., 1999; Fernàndez-Busquets et al., 2002).

Overall, the presented information indicates that archaeocytes perform characteristic functions of M1 bactericidal and M2 tolerogenic macrophages within signal organisms according to the situational context. Particularly, archaeocyte display an exceptional level of totipotency and autonomy (Zhang et al., 2003; Müller, 2006). We may speculate that archaeocytes execute important regulatory tasks in the sponge body and thus functionally precedes the role of neural and endocrine system.

We observe that as the complexity of multicellular organisms increases, the repertoire of functions performed by macrophage-like amoebocytes increases. This can be attributed to the need for a higher degree of regulation and maintenance of homeostasis or to the fact that specialized cells (in this case choanocytes) have taken over the original nutritional function of amoebocytes, thus providing macrophages with the opportunity to acquire additional diverse functions.

### Macrophage-like cells in animals with specialized tissues display rich repertoire of functions

Considering macrophage functions have diversified in animal evolution with the increasing complexity of the body, it is important to pay close attention to the macrophage-like plasmatocytes in *D. melanogaster*, a simple animal with clearly defined tissues and organs.

*Drosophila* is a simple, genetically tractable model organism, often used to model human diseases. Over a century of genetic and molecular biological research has led to many fundamental discoveries and a knowledge base that is unparalleled by any other invertebrate model used for biological research (Jennings, 2011). Research on the innate immune system of *Drosophila* has provided one of the major breakthroughs in immunology, the discovery of the Toll receptor and downstream immunity-related signaling cascade (Lemaitre et al., 1996). Since then, *Drosophila* has become a widely used model organism for research on host-microbe interactions, immune signaling pathways, wound healing, phagocytosis, clearance of apoptotic and damaged cells, tissue repair, immuno-metabolism, etc. (Razzell et al., 2011).

Most innate immune pathways known in mammals are highly conserved in *Drosophila*, including PRRs, second messengers, transcription factors, and effector molecules (Govind, 2008). Given that the conservation of immune pathways has been extensively described in many previous works, we mention them only briefly with emphasis on their evolutionary development and instead focus on immune-unrelated properties of plasmatocytes, such as their role in morphogenesis, regulation of metabolism, their tissue-specific roles, and their ability to phenotypically polarize.

Compared to basal clades of animals, the *Drosophila* immune system shows several significant advances. Firstly, the cellular branch of the immune system is represented by three distinct cell types with characteristic immunity-related functions. Crystal cells and lamellocytes are essential for the melanization reaction and the encapsulation of foreign objects that cannot be simply phagocytized, such as parasitoid eggs. Plasmatocytes are professional phagocytes that resemble macrophages in many of their properties (Gold and Brückner, 2015) with a high degree of molecular conservation of the underlying mechanisms (Melcarne et al., 2019).

The evolutionary novelty of the tunable immune response can be further documented by the variation of immune cascade activation following the recognition of different pathogens by PRRs on plasmatocytes. While fungi and Gram-positive bacteria elicit an immune response by activating the Toll receptor, Gram-negative bacteria predominantly activate the peptidoglycan receptor PGRP-LC and the downstream immune cascade IMD (De Gregorio, 2002). The components of the Toll and IMD immune cascades are highly conserved and show homology with downstream signaling from Toll-like receptors, NOD, GPCRs, and TNFR (Martinelli and Reichhart, 2005). Importantly, the diversity of immune-related signaling pathways enables the production of a cocktail of destructive effector molecules specifically tailored to the given pathogen, leading to an effective immune response while limiting immune-mediated damage to the host.

Moreover, the immune-related signaling pathways are accompanied by the production of various signaling factors that further influence other branches of the immune system and modify the function of other organs and tissues. Many of these factors can be denoted as true cytokines because their mammalian homologues are important regulators of the immune response, such as *unpaired3* (IL-6) or *eiger* (TNFα) (Vanha-aho et al., 2016).

In addition to their protective functions, plasmatocytes also have many macrophage-like properties essential for tissue homeostasis. They are responsible for clearing apoptotic, senescent, and damaged cells through efferocytosis and express various genes required for the remodeling of the ECM (Preethi et al., 2020). These abilities predispose them to play important roles in fundamental processes of multicellular organisms, such as embryonic morphogenesis, tissue healing, and regeneration. Indeed, plasmatocytes are essential for the patterning and developmental morphogenesis of the ventral nerve cord, intestine, heart, and skeletal muscle (Yarnitzky and Volk, 1995; Olofsson and Page, 2005).

However, the participation of plasmatocytes in embryonic morphogenesis can be disrupted by the production of danger signals. In an experimental model of laser-induced injury in the *Drosophila* embryo, plasmatocytes are attracted to the site of the wound by oxygen peroxide produced by the injured cells. Plasmatocytes infiltrating the wounded tissue clear the damaged cells and provide ECM components and growth factors necessary for tissue regeneration (Wood et al., 2002). Interestingly, the underlying mechanism of wound healing that includes transcription factors, actin organization, cell infiltration, and morphogenesis appears to be conserved between *Drosophila* and mammals at the molecular level (Belacortu and Paricio, 2011).

Although it has been well documented that *Drosophila* plasmatocytes can perform both bactericidal and healing functions, the question whether plasmatocytes adopt functional and metabolic polarization has not yet been satisfactorily answered. Upon bacterial infection in adult flies, plasmatocytes enter a state that closely resembles the pro-inflammatory polarization of mammalian macrophages. Plasmatocytes stimulated by streptococcal infection exhibit increased transcriptional activity of hypoxia-inducible factor 1α (HIF1α), a master regulator of metabolic reprogramming in mammalian M1 macrophages. The transcriptional program directed by HIF1α is required for the infection-induced increase in glycolytic flux, glucose consumption, and accelerated conversion of pyruvate to lactate in *Drosophila* plasmatocytes. This metabolic reprogramming, which closely resembles aerobic glycolysis in mammalian M1 macrophages, is essential for increased bactericidal activity of plasmatocytes and resistance of flies to bacterial infection (Krejčová et al., 2019). Transcriptomic data obtained in an independent experimental system indicate that metabolic rearrangement of plasmatocytes may be a general prerequisite for the bactericidal function of these cells (Ramond et al., 2020).

Plasmatocyte polarization that resembles mammalian M2 macrophages has been observed in an experimental model of retinal tissue injury. In this scenario, plasmatocytes infiltrate the wound and promote tissue healing, presumably by increasing the expression of arginase, an enzyme promoting the conversion of arginine to ornithine necessary for tissue regeneration (Neves et al., 2016), which is the hallmark of M2 macrophages polarization in mammalian macrophages (Rath et al., 2014). Whether plasmatocytes adopt an M2-like phenotype during other situations, such as during efferocytosis or wound healing, remains to be investigated.

The idea of functional diversification of plasmatocytes in *Drosophila* also finds support in single-cell transcriptomic analysis of *Drosophila* immune cells. The available data suggest that despite the morphological uniformity, plasmatocytes consist of more than ten distinct subpopulations that differ markedly in their expression pattern and expression of characteristic markers (Cho et al., 2020; Tattikota et al., 2020). Albeit, functional confirmation of these observations is currently lacking. In depth analysis of single-cell data revealed that a particular population of plasmatocytes express a substantial number of genes related to lipid metabolism, lipid catabolism, and sphingolipid processing, indicating certain adipocyte features are also present in plasmatocytes (Tattikota et al., 2020).

Of the many situations in the fly life cycle, the most important metabolic role of plasmatocytes is, arguably, during metamorphosis. During the transition of the larva into the adult, the lymph gland is broken down, and plasmatocytes are released into the circulation (Kharrat et al., 2022). The vast majority of larval tissues undergo extensive histolysis, and adult tissues form *de novo* from imaginal discs. However, the energy accumulated during the larval life stage must be transferred to the adult (Merkey et al., 2011). Thus, cells that are no longer needed are removed during metamorphosis by plasmatocytes infiltrating the histolysis-undergoing tissues. Within a short period of time, thousands of cells must be efferocytosed and the building material recycled into a suitable, reusable form (Storelli et al., 2019). In this situation, cells predisposed to efficient processing of lipids and sphingolipids may be highly desirable.

Plasmatocytes not only serve as metabolically active cells *per se*, but also significantly regulate the metabolism of other tissues. During bacterial infection, plasmatocytes produce the factor called *Imaginal morphogenesis protein-Late2* (*ImpL2*), which reduces insulin signaling in the fat body. In turn, the fat body produces lipoproteins and carbohydrates that replenish activated immune cells (Krejcova et al., 2019). Thus, plasmatocytes orchestrate metabolic homeostasis and nutrient redistribution during the stress response. Their role in regulating systemic metabolism has been further documented in flies fed a high-fat diet. During excessive energy intake, plasmatocytes exposed to excessive lipids secrete IMPL2, leading to increased circulating glucose levels. Therefore, suppression of *ImpL2* in plasmatocytes improves metabolism in obese flies (Morgantini et al., 2019). The pro-inflammatory effect of lipids on plasmatocytes was confirmed in an independent study. Plasmatocytes exposed to excessive amounts of lipids engulf the lipids through the activity of the scavenger receptor *croquemort*, which is homologous to mammalian CD36. Lipid accumulation in the plasmatocyte cytosol leads to increased production of the cytokine *unpaired3* (*upd3*) and systemic attenuation of insulin signaling *via* JAK/STAT signaling (Woodcock et al., 2015). Interestingly, *upd3* production by plasmatocytes may have an adaptive significance in addition to its pathological role analogically to *ImpL2*. It has been shown experimentally that sustained production of UPD3 by plasmatocytes is required for the regular distribution of lipids between tissues in the body and that a missing UPD3 signal leads to lipid accumulation in muscles (Kierdorf et al., 2020).

It is unclear whether *Drosophila* has tissue macrophages as we know them in mammals. Functionally, the cells that most closely resemble the concept of tissue macrophages in *Drosophila* are cells that can be functionally considered as microglia. Microglia are the resident macrophages of the mammalian central nervous system (CNS) and are responsible for the immune protection of neurons and elimination of toxic and harmful substances, and for the maintenance, neuronal pruning, and proper functioning of synapses in CNS (Lee et al., 2021).

Glial cells in *Drosophila,* similar to their mammalian counterpart, form the brain-blood barrier and maintain homeostasis of the CNS of flies. Although no plasmatocytes reside in the *Drosophila* brain under physiological conditions, glial cells display molecular parallels regarding their phagocytic receptors *six microns under* (*simu*) and *draper* (*drpr*) (Kim et al., 2020). Glial cells expressing *Simu* and *drpr* are required for clearance of the impaired neurons and neuronal debris, and the lack of expression these receptors leads to neurodegeneration (Elliott and Ravichandran, 2008). Moreover, a microglia-like glial subtype called MANF (Mesencephalic Astrocyte Derived Neurotrophic Factor) immuno-reactive cells has been described in the *Drosophila* brain during metamorphosis under certain conditions. These cells are extremely rich in lysosomes and express *drpr* (Stratoulias and Heino, 2015). In addition, cortex glia and ensheathing cells are non-professional phagocytes engulfing apoptotic cells during the development of the nervous system and degenerating axons, respectively (Doherty et al., 2009; Kurant, 2011).

Since insects lack the adaptive immune system that evolved 500 million years ago in jawed fish, they must rely solely on innate immunity. Non-etheless, it has been described that the innate immune system can also be “trained”, and display certain memory traits. The phenomenon of “innate immune memory” was proposed by Netea et al, (2020), who conducted this research on mammalian models. This concept has also been addressed in *Drosophila*. It has been documented that fruit flies display enhanced survival of streptococcal infection if re-encountered by an otherwise lethal dose of the same bacteria and that this protective mechanism lies in the action of phagocytes and the Toll signaling pathway (Pham et al., 2007). However, such protection could not be invoked against all the bacteria examined.

As evidenced by advances made in recent years, plasmatocytes in *Drosophila* perform a strikingly wide range of roles that encompass the functional repertoire of M1 and M2 macrophages. In addition, experimental data demonstrate that plasmatocytes are capable of entering different polarization phenotypes over time. Moreover, several lines of evidence suggest that plasmatocytes are not a uniform population and consist of many distinct subpopulations of plasmatocyte phenotypes. Particularly, their ability to regulate the metabolism of other tissues *via* signaling factors might be of interest. In terms of the pathological role of mammalian macrophages, it is an interesting observation that exposure of plasmatocytes to excessive amounts of lipids may lead to macrophage polarization, reminiscent of Mox polarization in mammalian macrophages. Whether *Drosophila* possesses a functional analogy to mammalian tissue-resident macrophages remains to be determined.

## Macrophage functional versatility as a legacy of animal origin

**FIGURE 3:**
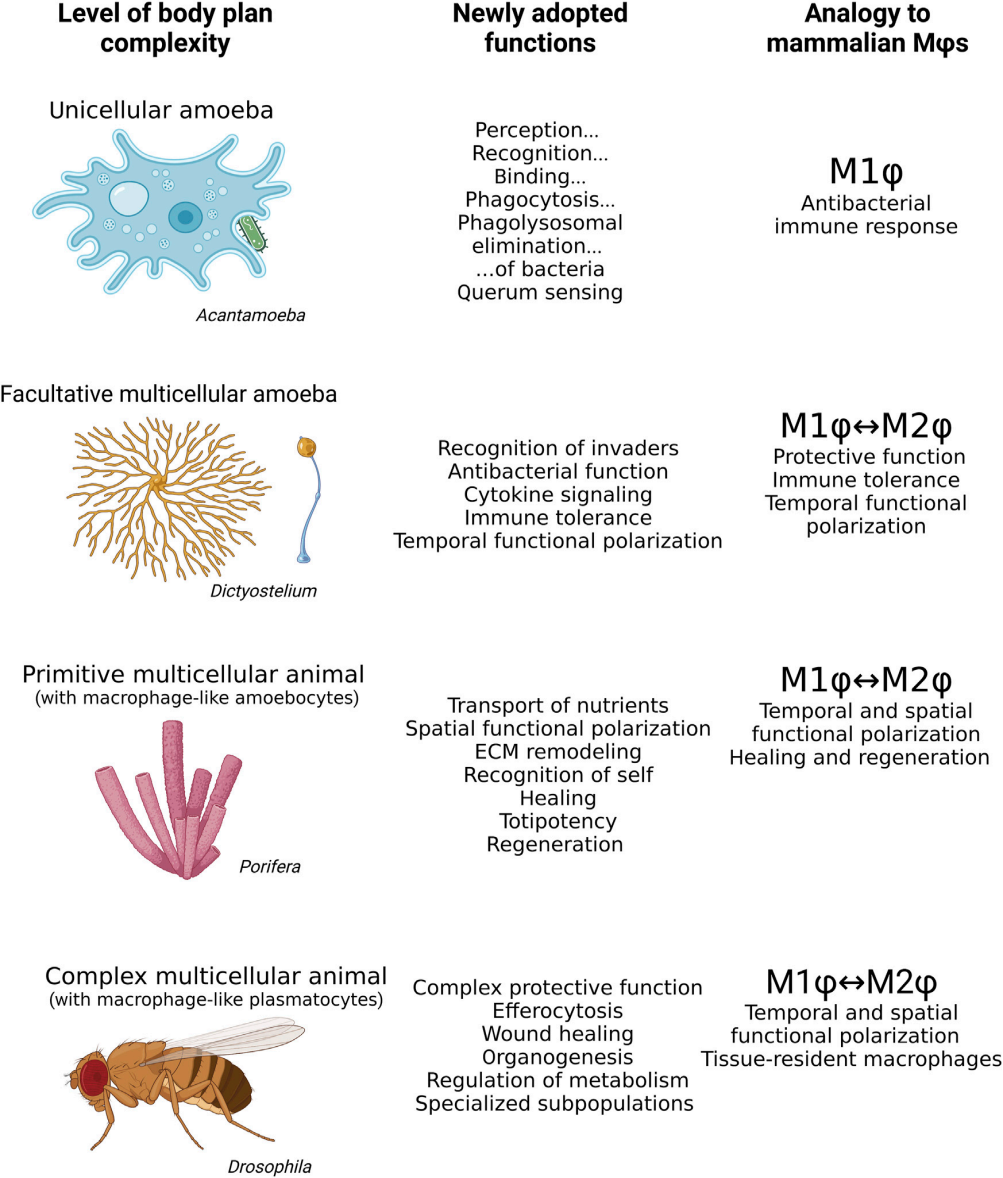
Mammalian macrophages share many features with *Acanthamoeba, Dictyostelium*, archaeocytes in *Porifera,* and plasmatocytes in *Drosophila*. While the bacteria hunting unicellular *Acanthamoeba* resembles the M1 polarization phenotype of mammalian macrophages, *Dictyostelium* exhibits M1 or M2 features of mammalian macrophages depending on its life stage. The archaeocytes of aquatic sponges have both M1 and M2 macrophages within a single organism, phenotypically responding to situation context rather than life stage. Plasmatocytes of *Drosophila* exhibit a wide range of highly specialized roles in the organism in addition to M1 and M2 polarization. *M*ϕ*s, macrophages.*

In the previous paragraphs, we have discussed how the rising complexity of body plans corresponds with the adoption of crucial macrophage features (Figure 3). Nevertheless, the question of why macrophages are predisposed to exceptional functional versatility remains to be addressed.

Recently, the theoretical concept of the origin of multicellular animals has been revisited. It is generally accepted that amoebocytes represent the most ancestral cell type of all *Holozoa*. Primitive facultative multicellular animals consisted of a cluster of a few cell types, temporally forming multicellular colonies (Ros-Rocher et al., 2021).

The observations from *Dictyostelium* and *Porifera* indicate that amoebocytes, the last common ancestor of multicellular animals, represent the ancestral super-ordinated cells that must have been widely distributed and capable of functionalities performed in more complex animals by specialized tissues and organs. They were most likely able to differentiate into all other cell types, control their number, and govern protective, nutritional, regulatory, and homeostatic functions. Therefore, we expect these cells to be already highly functionally versatile with a certain level of plasticity and autonomy.

Given that macrophage-like amoebocytes represent an archetypal cellular type in animals (Cavalier-Smith, 2017), the second cell type commonly diversified in early multicellular organisms are cells specialized for acquiring nutrients from the environment (Sogabe et al., 2019). We can hypothesize that as amoebocytes no longer needed to obtain nutrients for themselves, they evolved to perform other functions in the multicellular body. However, the differentiation of individual specialized cells imposed the requirement to evenly distribute resources, coordinate the function of individual cells, and maintain homeostasis in response to changing external biotic and abiotic factors (Bich et al., 2019). These requirements demand a certain level of regulation, and before the development of the circulatory, endocrine, and neuronal systems, the amoebocytes were predisposed to perform such functions (Dyakonova, 2022).

We believe that macrophage functional versatility may be a heritage of their origin in unicellular and early multicellular animals, where the universality of macrophage-like amoebocytes was essential for resistance to different types of environmental and biological stress. Over millions of years of evolution from amoebocytes to macrophages, macrophage-like cells have taken advantage of their initial versatility and gradually achieved their full functional repertoire along with the increasing complexity of the animal body. Although we might assume that the functional variability of macrophages would decrease with the emergence of organ systems, the opposite is true. Every tissue in the mammalian body contains a population of tissue-resident macrophages that often perform highly specialized functions (Mass et al., 2016). Collectively, maintaining functionally versatile amoeboid cells that can easily change their functional repertoire to suit emerging needs seems to be an adaptive strategy.

## The origin of macrophage functions may explain their pathological effect in mammals

In general, it can be assumed that the acquisition of new functions of macrophages in evolution can be achieved by changing their original archetypal role and adapting it to the current context (Brosius, 2019). This can be documented, for instance, by a functional shift from mechanisms evolved to hunt bacteria to an antibacterial protective role of macrophages. The mechanism required by unicellular amoeboid cells to identify, approach, phagocytose, and digest bacteria in the phagolysosome for nutritional reasons was later shown to be advantageous for macrophages in multicellular organisms for protection against pathogenic bacteria (Hartenstein and Martinez, 2019). Another such example is the rich repertoire of genes originally used in the unicellular ancestor of animals for amoeboid crawling and attachment to surface structures, which evolved into a broad repertoire of surface receptors and adhesion molecules used by macrophages in multicellular organisms for sensing surrounding tissues and motility (Hynes and Zhao, 2000).

Thus, many signaling pathways in macrophages and other myeloid cells may carry remnants of their evolutionary origin without retaining their initial adaptive function in a complex multicellular organism. Such vestigial molecular relationships may underlie the pathological behavior of macrophages. For example, the folate receptor, formyl-peptide receptor, or cAMP signaling represent the shift of adaptive functions originally developed in macrophage-like amoebocytes to their pathological effect in macrophages. We believe that many analogous comparisons can be found when applying this perspective to human pathologies.

Folate, a secondary metabolite of bacteria, is a potent chemoattractant for amoeboid vegetative cells of *Dictyostelium* (Driel, 1981). The folate gradient is perceived *via* a G-protein coupled folate receptor at nanomolar concentrations and leads to the activation of chemotaxis and machinery required for phagocytosis and bacterial processing in the phagolysosome (Pan et al., 2016). Interestingly, increased expression of folate receptors are a hallmark of pro-inflammatory mammalian macrophages (Steinz et al., 2022). In particular, folate receptor *β* (FR-β) has been identified as a specific surface receptor for highly pro-inflammatory macrophages, such as those found in the synovial tissue of arthritic patients, in atherosclerotic plaques, or in pulmonary fibrosis (Chandrupatla et al., 2019). Activation of macrophage FR-β leads to their pro-inflammatory polarization and production of cytokines that further perpetuate the chronic inflammatory state. Inhibition of the folate receptor has thus been recognized as a possible avenue for treating arthritis and atherosclerosis, making FR-β agonist Methotrexate the first-choice treatment for these diseases (Xia et al., 2009).

Similar functional dualism can be observed for other GPCRs carried by mammalian myeloid cells, such as the formyl peptide receptor (FPR) abundantly expressed by macrophages and neutrophils (Chen et al., 2017). Activation of FPR serves as a potent signal leading to enhanced directional motility, the production of ROS, the release of pro-inflammatory cytokines, and acceleration of phagocytic and bactericidal machinery (Liang et al., 2020). Since formyl peptides are released by bacteria as their secondary metabolite, the response mediated *via* the FPR receptor is important for resistance to bacterial pathogens (Dorward et al., 2015). However, under stress conditions, formyl peptides are released from the mitochondria of stressed and damaged tissues, leading to infiltration of the affected tissue by macrophages and neutrophils, which induce inflammation even under sterile conditions (Wenceslau et al., 2013). Therefore, excessive activation of FPR on macrophages and neutrophils underpins the progressive development of many human inflammatory diseases, such as neurodegeneration, cardiovascular diseases, and pulmonary fibrosis (Trojan et al., 2020; Caso et al., 2021).

In both cases, inadequate activation of receptors, initially designed to detect bacterial secondary metabolites and tracking bacteria in the environment, causes pathology in a complex multicellular organism, where their activation can occur even under sterile conditions (Lu et al., 2021).

Non-etheless, the repurposing of ancestral signaling is not limited to bacterial detection and localization mechanisms. As described previously, metabolically stressed vegetative amoebae of *Dictyostelium* produce cAMP as a potent aggregation chemoattractant (Singer et al., 2019). Sensing of extracellular cAMP leads to the activation of stress-related cellular pathways, remodeling of cellular metabolism, and epigenetic remodeling, resulting in a transition to multicellularity, increased production of ECM components, and tolerogencity (Wang S. Y. et al., 2021). Interestingly, many lines of evidence suggest that extracellular cAMP (ex-cAMP) strongly effects the recruitment and reprograming of monocytes and macrophages and induces efferocytosis of damaged or exhausted surrounding cells (Negreiros-Lima et al., 2020). Exposure of monocytes to ex-cAMP enhances the production of cytokines with known anti-inflammatory effects, such as IL-6 and IL-10, and ameliorates response to pro-inflammatory stimuli (Sciaraffia et al., 2014).

cAMP in the extracellular space is cleaved by ectonucleotidases to extracellular adenosine and sensed by the adenosine receptor abundantly expressed by macrophages and other myeloid cells (Haskó and Pacher, 2012). Adenosine and cAMP are released from damaged, hypoxic, and metabolically stressed tissues. Activation of adenosine receptors causes potent anti-inflammatory effects and plays an essential role in tissue regeneration and maintenance of tissue homeostasis (Pasquini et al., 2021). Interestingly, cAMP is an important secondary messenger in mammalian immune cells that activates identical downstream cascades in *Dictyostelium* amoebocytes, leading to the inhibition of NFĸB and activation of anti-inflammatory tolerogenic polarization (Tavares et al., 2020).

Thus, we can assume that cAMP signaling, which appeared in evolution at the origin of multicellular animals, may play an adaptive role in the immune system up to the present day. However, adopting a tolerogenic program through the activation of adenosine and cAMP signaling also has a role in pathology. Increased adenosine and cAMP production by metabolically demanding and often hypoxic neoplastic tumors leads to the induction of tolerogenic polarization in surrounding immune cells (Strakhova et al., 2020). Hence, tumor-associated macrophages often promote tumor growth, instead of elimination, by providing nutrients and growth factors and promoting vascularization (Moeini and Niedźwiedzka-Rystwej, 2021).

Thus, the repurposing of the features of the macrophage ancestors may be adaptive, as evidenced by protection against pathogenic bacteria, but may contribute to the development of many pathologies.

## Discussion

The hypotheses we present here are speculative, convincing evidence that documents events that took place in the distant past in evolution is limited. However, this may change significantly with the growing list of organisms with fully sequenced genomes and well-annotated transcriptomes. Many of these newly sequenced species provide information allowing speculation regarding the nature of the last unicellular and first multicellular ancestors of animals. These data provide evidence of genes that were prerequisites for the emergence of multicellularity and the development of advanced multicellular body structures. An interesting example of such an approach can be found in the work of Ros-Rocher and colleagues, and it is feasible that analogous analyses can yield valuable information in the future (Ros-Rocher et al., 2021). Regarding the origin of macrophage functional versatility, the effort requires tracing macrophage characteristic features in evolution. Recent work conducted by Nagahata and colleagues, which, on the genetic level, supports the hypotheses that many functions typical of bactericidal macrophages evolved from a common ancestor of animals, and that many characteristic macrophage features are adaptations of free-living unicellular bacterivorous amoebae (Nagahata et al., 2022; Rayamajhee et al., 2022).

The majority of macrophage-like features that are observed in amoebae resemble those of bactericidal (M1) macrophages. This indicates that the bactericidal macrophage polarization represents an ancestral polarization type and the protective function of macrophages evolved from hunting microbes for nutritional reasons (Desjardins et al., 2005; Hartenstein and Martinez, 2019).

Inspired by the currently revised theory of the origin of animal multicellularity (Brunet and King, 2017), we believe that amoebocytes, as the ancestral type of animal cells, play a central role in the origin of multicellularity. Amoebocytes display several features that may be considered prerequisites for the emergence of multicellularity, such as the ability to deposit and remodel ECM, remove senescent and damaged cells, respond to various signals, regulate the function of other cells by signaling factors, and recognize genetically related cells in the colony (Misevic, 1999). These features are required by multicellular organisms and also resemble the characteristics of healing (M2) macrophages. Therefore, we suggest that along with the emergence of multicellular animals, the macrophage-like amoeboid cells acquired macrophage-like properties characteristic of M2 macrophages.

Given that the last common ancestor of animals likely switched between free-living and colonial life stages during its life cycle in response to extrinsic cues (Brunet et al., 2021), it is possible that the macrophage-like amoebocytes had the capacity for phenotypic polarization in a context-dependent manner, before the emergence of multicellularity. While the wild-type social amoeba shows features observed predominantly in M1 macrophages, amoebocytes participating in colonial life stages have changed their biology and acquired features characteristic predominantly of M2 macrophages.

We can hypothesize that the divergence of macrophage functions in emerging multicellular organisms was driven by the differentiation of specialized cell types for obtaining nutrition, the growing need for harmonizing force, and the recognition of cellular identity. The diversification of the functional repertoire of macrophages in primitive multicellular organisms (*Porifera*) suggests that, together with the increasing complexity of body plans, there is a need for polarization of macrophage-like archaeocytes into both phenotypes within a single organism, with the polarization phenotype depending on context rather than life stage (Degnan et al., 2015). We may hypothesize that the regulatory role of macrophage-like amoebocytes precedes the function of the endocrine and nervous systems in primitive multicellular organisms, indicating a superior regulatory role of amoebocytes over other cells in the body.

Upon the evolution of complex multicellular organisms, macrophages expanded their functions, reaching their full potential, participating in development, organogenesis, immune protection, self-recognition, tissue and metabolic homeostasis maintenance, and many tissue- and context-specific tasks (Mase et al., 2021). It is likely that these functions will be revealed in future research in virtually all complex multicellular animals.

We present a perspective of evolutionary biology, combined with knowledge from modern biomedical research. Both approaches can be mutually inspiring in future research on the biology of macrophage-like cells. One critical feature which contributes to the functional versatility of macrophages is their ability to adopt distinct metabolic polarization phenotypes, which are determined by epigenetic modifications and activation of specific signaling cascades. Therefore, investigating polarization phenotypes of unicellular and facultative multicellular relatives of true animals would be of interest. A seminal study addressing this was performed on *Dictyostelium*, carried out in the laboratory of Erika Pearce, one of the leading scientists working on macrophage immuno-metabolism (Kelly et al., 2021). This work demonstrated a link between cell metabolism and the transition to the multicellular stage. In addition, the traits of early relatives of animals can be explored for their significance to mammalian macrophage biology. One such example is the potentially conserved nutritional role of macrophage-like amoebocytes in *Dictyostelium* and sponges in the macrophages of higher animals and humans.

In addition, we believe that understanding the role of ancestral macrophage-like cells may help to understand the biology of mammalian macrophages and possibly discover new functions. Given the ancestral origin of macrophage functions, some difficult-to-understand pathological behaviors of macrophages can be explained by the activation of ancient vestigial functions that may appear counterintuitive in a specific context in a complex multicellular body. We believe that this perspective may shed new light on the function and pathogenesis of macrophages in animals and humans.

## Data Availability

The original contributions presented in the study are included in the article/supplementary material, further inquiries can be directed to the corresponding authors.
